# Automated Koos Classification of Vestibular Schwannoma

**DOI:** 10.3389/fradi.2022.837191

**Published:** 2022-03-10

**Authors:** Aaron Kujawa, Reuben Dorent, Steve Connor, Anna Oviedova, Mohamed Okasha, Diana Grishchuk, Sebastien Ourselin, Ian Paddick, Neil Kitchen, Tom Vercauteren, Jonathan Shapey

**Affiliations:** ^1^School of Biomedical Engineering and Imaging Sciences, King's College London, London, United Kingdom; ^2^Department of Neuroradiology, King's College Hospital, London, United Kingdom; ^3^Department of Radiology, Guy's Hospital, London, United Kingdom; ^4^Department of Neurosurgery, King's College Hospital, London, United Kingdom; ^5^Queen Square Radiosurgery Centre (Gamma Knife), National Hospital for Neurology and Neurosurgery, London, United Kingdom; ^6^Wellcome/Engineering and Physical Sciences Research Council (EPSRC) Centre for Interventional and Surgical Sciences, University College London, London, United Kingdom; ^7^Department of Neurosurgery, National Hospital for Neurology and Neurosurgery, London, United Kingdom

**Keywords:** vestibular schwannoma, classification, segmentation, deep learning, artificial intelligence

## Abstract

**Objective:**

The Koos grading scale is a frequently used classification system for vestibular schwannoma (VS) that accounts for extrameatal tumor dimension and compression of the brain stem. We propose an artificial intelligence (AI) pipeline to fully automate the segmentation and Koos classification of VS from MRI to improve clinical workflow and facilitate patient management.

**Methods:**

We propose a method for Koos classification that does not only rely on available images but also on automatically generated segmentations. Artificial neural networks were trained and tested based on manual tumor segmentations and ground truth Koos grades of contrast-enhanced T1-weighted (ceT1) and high-resolution T2-weighted (hrT2) MR images from subjects with a single sporadic VS, acquired on a single scanner and with a standardized protocol. The first stage of the pipeline comprises a convolutional neural network (CNN) which can segment the VS and 7 adjacent structures. For the second stage, we propose two complementary approaches that are combined in an ensemble. The first approach applies a second CNN to the segmentation output to predict the Koos grade, the other approach extracts handcrafted features which are passed to a Random Forest classifier. The pipeline results were compared to those achieved by two neurosurgeons.

**Results:**

Eligible patients (*n* = 308) were pseudo-randomly split into 5 groups to evaluate the model performance with 5-fold cross-validation. The weighted macro-averaged mean absolute error (*MA-MAE*), weighted macro-averaged F1 score (*F*_1_), and accuracy score of the ensemble model were assessed on the testing sets as follows: *MA-MAE* = 0.11 ± 0.05, *F*_1_ = 89.3 ± 3.0%, *accuracy* = 89.3 ± 2.9%, which was comparable to the average performance of two neurosurgeons: *MA-MAE* = 0.11 ± 0.08, *F*_1_ = 89.1 ± 5.2, *accuracy* = 88.6 ± 5.8%. Inter-rater reliability was assessed by calculating Fleiss' generalized kappa (k = 0.68) based on all 308 cases, and intra-rater reliabilities of annotator 1 (k = 0.95) and annotator 2 (k = 0.82) were calculated according to the weighted kappa metric with quadratic (Fleiss-Cohen) weights based on 15 randomly selected cases.

**Conclusions:**

We developed the first AI framework to automatically classify VS according to the Koos scale. The excellent results show that the accuracy of the framework is comparable to that of neurosurgeons and may therefore facilitate management of patients with VS. The models, code, and ground truth Koos grades for a subset of publicly available images (*n* = 188) will be released upon publication.

## Introduction

Vestibular Schwannoma (VS) is a benign, slow growing tumor that develops in the internal auditory canal which passes from the inner ear to the brain. The tumor results from an abnormal multiplication of Schwann cells within the insulating myelin sheath of the vestibulocochlear nerve. It can impair hearing and balance but can become life-threatening if it compresses the brain stem or other cranial nerves. It has been estimated that approximately 1 in 1,000 people will be diagnosed with a VS in their lifetime (1); however, the incidence of VS has been noted to be rising as a result of improved magnetic resonance imaging (MRI) image quality that facilitates the detection of smaller VS (2).

The type of treatment is typically based on the tumor size and its impact on adjacent brain structures. In a study from 2006, it was observed that most patients had exhibited no significant tumor growth over a mean observation time of 3.6 years (2). This encouraged a shift toward conservative management, especially for small intrameatal tumors (3). Extrameatal tumors are more likely to exbibit growth and to impair the patient's wellbeing. Such tumors are more often treated with radiosurgery, radiotherapy, or microsurgery. Surgery is favored for larger tumors exhibiting mass effect.

**Figure 1:**
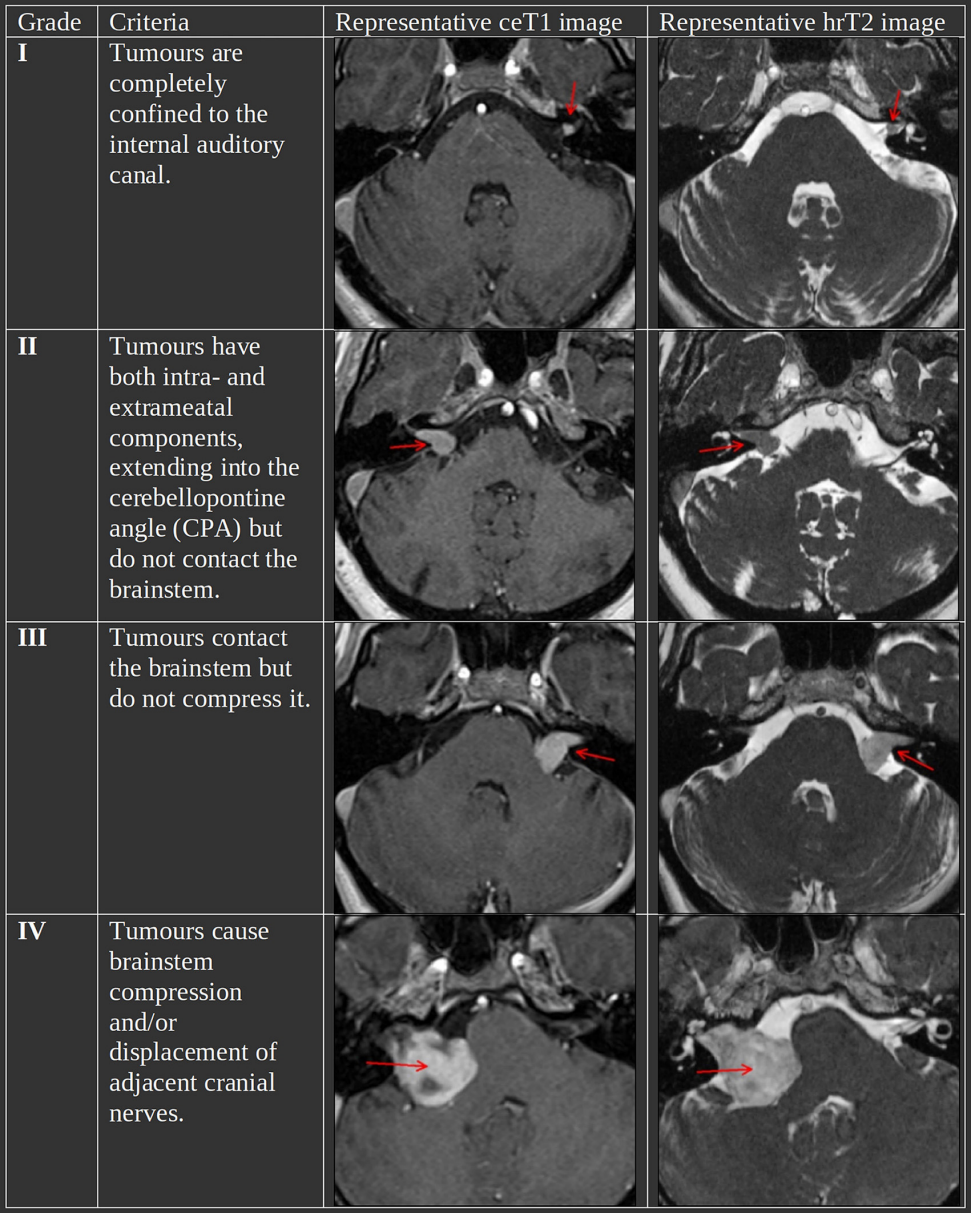
The Koos scale with representative ceT1 and hrT2 images. The images in each row are from the same subject and scan session. Red arrows in the MR images indicate the tumor.

The Koos grading scale is a classification system for VS that captures many of the characteristics that treatment decisions are typically based on (4). Figure 1 summarizes the criteria and shows representative MR images for each grade. In a recent study, it has been shown to be a significantly reliable means to characterize VS by evaluating the inter-and intra-rater reliability based on 40 and 10 subjects, respectively (5). Furthermore, in clinical practice, it is regularly determined among other metrics such as the maximal linear (extrameatal) dimension in order to decide on a treatment plan (6, 7).

Artificial Intelligence (AI)-driven clinical support tools have the potential to improve patient outcomes and experience by the standardization and personalization of VS treatment (8). In recent years, particularly with the advent of deep learning, AI algorithms have been developed to automate time-consuming and repetitive tasks to reduce the workload for clinical staff. Previous work has been focused on performing automatic segmentation of VS using deep learning frameworks (9, 10) and achieved high segmentation accuracy on a large publicly available dataset of MR images (11). However, the problem of VS classification has not been addressed, yet.

Classification of other brain tumors has been performed in different settings. A frequently addressed task is the classification of tumor types, such as Meningioma, Glioma and Pituitary (12–14), or Astrocytoma, Glioblastoma, Oligodendrogloma (15). Other tasks that received much attention are the classification of glioma grades (I-IV) (16, 17) and classification of benign and malignant tumor stages (I-IV) (18, 19). These tasks have in common that classification from MRI images can benefit from characteristic textural features and modality dependent characteristic appearance of tumor types and grades. In contrast, Koos grades of VS are defined by tumor size, location, and compression of adjacent brain structures while tumor texture and modality dependent appearance are less relevant. Therefore, we suggest that an accurate segmentation label mask of the tumor and relevant adjacent brain structures provides sufficient information for Koos classification.

In this work, we introduce the first Machine Learning framework for Koos classification in an automated pipeline. This work introduces the following contributions:

1. Instead of performing classification directly on the images, we propose a 2-stage approach that achieves classification after an initial segmentation stage.
2. After defining a procedure to identify adjacent brain structures that are important for Koos grading, we develop a fully automated segmentation model that segments not only the VS but also these structures. Bronze standard segmentations for these structures were obtained using an accurate but time-consuming segmentation framework (20) and then used to train a state-of-the-art deep learning framework (21).
3. For the second stage, we propose two complementary approaches to perform Koos classification. One approach is based on data-driven features automatically extracted with a convolutional neural network (CNN) (22). The other approach relies on handcrafted features extracted from the segmentation. These features were specifically designed for our task and used as input for a Random Forest (23). Finally, the approaches are combined in a model ensemble that can leverage the strengths of each constituent model to enhance classification accuracy.
4. Extensive experiments on a large dataset (*n* = 308) demonstrate the performance of our 2-stage approach. Our approach outperforms image-based classification methods. Higher accuracy is obtained by using the automatic segmentation of the identified adjacent structures. Our approach achieves a statistically equivalent performance in comparison to a neurosurgeon.

## Materials and Methods

### Description of Study Data

#### Ethics Statement

This study was approved by the NHS Health Research Authority and Research Ethics Committee (18/LO/0532). Because patients were selected retrospectively and the MR images were completely anonymised before analysis, no informed consent was required for the study.

#### Study Population

Imaging data from consecutive patients with a single sporadic VS treated with gamma knife (GK) stereotactic radiosurgery (SRS) on the day of the image acquisition between October 2012 and March 2021 were screened for the study. All adult patients older than 18 years with a single unilateral VS were eligible for inclusion in the study, except for patients who had previously undergone operative surgical treatment. Out of 384 patients, 308 patients (males/females 137:171; median age 57 years, IQR 50–67 years) met these initial inclusion criteria. All patients had MRI studies performed on a 1.5T scanner (Avanto Siemens Healthineers), including ceT1 MRI acquired with in-plane resolution/matrix size of 0.8 × 0.8 mm/256 × 256 or 0.4 × 0.4 mm/512 × 512, and slice thickness of 1.0–1.5 mm [TR (repetition time) 1,900 msec, TE (echo time) 2.97 msec, TI (inversion time) 1,100 msec] and an hrT2 MRI with either a Constructive Interference Steady State (CISS) sequence (in-plane resolution/matrix size/TR/TE = 0.47 × 0.47 mm/448 × 448/9.4 msec/4.23 msec) or a Turbo Spin Echo (TSE) sequence (in-plane resolution/matrix size/TR/TE = 0.55 × 0.55 mm/384 × 384/750 msec/121 msec) and slice thickness of 1.0–1.5 mm. We randomly assigned the final 308 patients into 5 non-overlapping groups for 5-fold cross-validation while ensuring that all cases of the same Koos grade were evenly distributed across the groups (stratified random sampling).

Out of the 308 patients, the imaging data of 188 patients are part of a publicly available dataset (11). The other 120 patients' imaging data are part of an extension of this dataset, acquired with the same scanner and protocol. The extension is currently not publicly accessible, because it forms the test set of a new challenge for Cross-Modality Domain Adaptation for Medical Image Segmentation (crossMoDA) (https://crossmoda-challenge.ml).

#### Ground Truth Segmentations

##### Vestibular Schwannoma Segmentation

To enable automatic segmentation of the tumor at inference time, a model was trained based on manual segmentations of VS in the training set. The segmentations were performed in consensus by the treating team, consisting of a consultant neurosurgeon (RB or NK) and a physicist (IP or AD) based on both the ceT1 and hrT2 images. The GK planning software (Leksell GammaPlan) offers a semi-automated segmentation method which was used to perform the manual segmentations in each axial image slice.

##### Brain Structure Segmentation

To supervise the training process of the segmentation network, bronze standard segmentations of brain structures adjacent to the VS were also provided. Rather than relying on time-consuming manual segmentations, we employed the GIF algorithm which automatically creates a high resolution parcellation of 160 distinct brain structures based on T1-weighted MR images (20). A quality check on segmentations of 10 randomly selected images was performed by JS. GIF itself relies on a database of 30 T1-weighted MR images and corresponding manual parcellations. In addition to the ceT1 image, a mask of the manual VS segmentation was passed as an input to the GIF algorithm which was done to exclude the voxels belonging to the VS from the automated parcellation process. Processing a single image with GIF takes ~5 h on an Intel core i9 CPU which prohibits its clinical deployment. Hence, in our pipeline, GIF is only applied to produce the segmentation ground truth which is used to supervise the training of the segmentation model.

#### Ground Truth Koos Grades

Two neurosurgeons with 5–10 years' experience (MO and AO) individually assigned Koos grades to all patients using both available modalities. Each annotator was briefed and provided with images and definitions of the Koos grading system as provided by Erikson et al. (5). This data was acquired to assess inter-rater agreement and to compare the performance of our automated pipeline to that of human annotators. Furthermore, to assess intra-rater reliability, the dataset presented to both human annotators included randomly selected duplicates of 15 pairs of scans (ceT1 and hrT2) which were presented to the human annotators after they had assigned a Koos grade to all 308 patients.

A second team of experts including a consultant neuroradiologist (SC) and a consultant neurosurgeon (JS) determined in consensus the Koos grade ground truth for cases on which the two other human annotators disagreed. If the two human annotators agreed, the Koos grade assigned by them was assumed as the ground truth. The manual VS segmentations and Koos grades were subsequently treated as the ground truth for the supervised training of the neural networks and Random Forest and for the evaluation of the predicted VS segmentations and Koos grades.

### AI Framework for Automated Koos Classification

**Figure 2:**
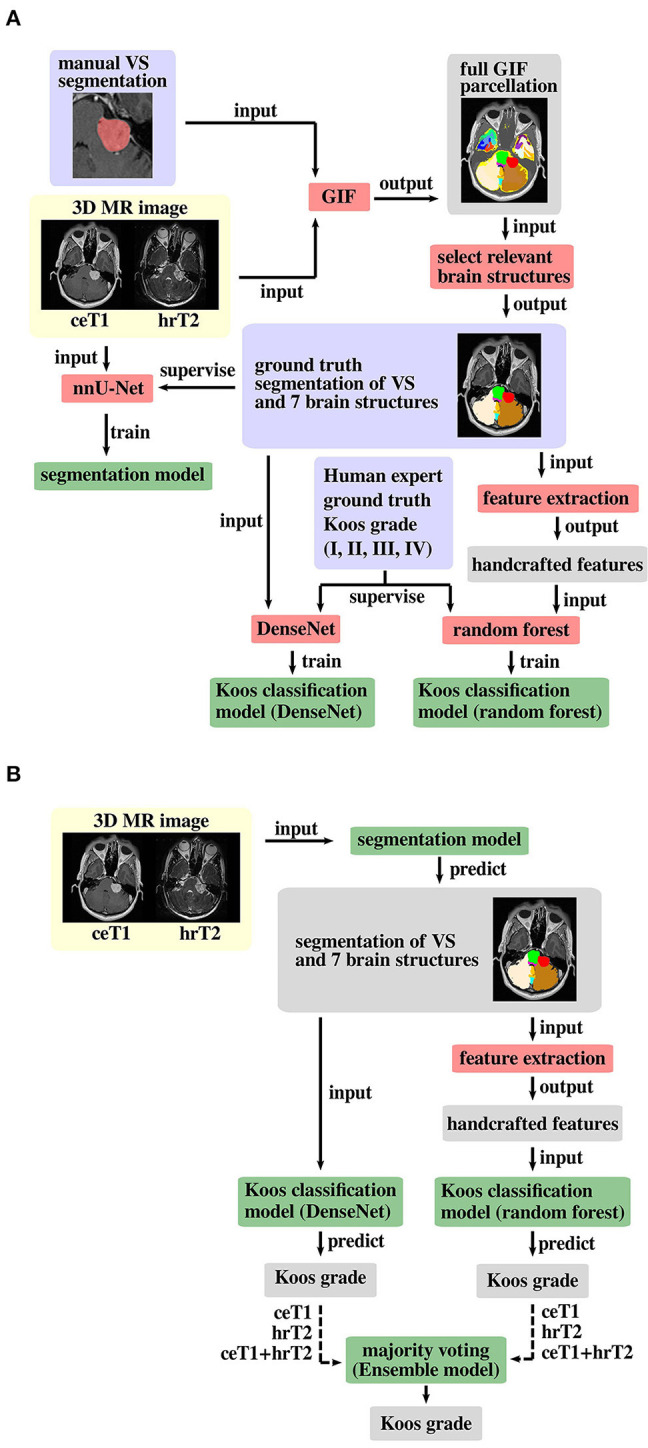
Proposed pipeline for **(A)** model training and **(B)** inference. The background colors indicate pre-processed MR images (yellow), algorithms/operations (red) applied to input data, ground truth data (blue) that is used to supervise the model training process, intermediate output data (gray) and trained models (green). Training of both Koos classification models is only required for an ensemble model of DenseNet and Random Forest, otherwise either model can be applied individually, and the other model's pipeline branches can be omitted for training and inference. The dashed arrow lines in the inference pipeline indicate that the whole pipeline (including training) must be run with each of the three different input types to obtain three Koos grade predictions per branch.

A schematic representation of the proposed pipeline is shown in Figure 2. The first stage of the training pipeline comprises two key steps: GIF to produce the segmentation ground truth and nnU-Net (16, 21) to train the segmentation model. In the second stage of the pipeline, one branch can be selected to train either the DenseNet (22) classification model, or the Random Forest (23) classification model for Koos classification, or both models if an ensemble model is used. The Random Forest branch starts with a handcrafted feature extraction step not found in the DenseNet branch. During inference, the segmentation and classification models are applied sequentially to predict the Koos grade.

#### The Segmentation Network

In the first stage of the pipeline, a Convolutional Neural Network (CNN) was applied to automatically segment the VS and brain structures. The process used to select the relevant brain structures is explained in section Selection of Relevant Brain Structures. For this step we employ nnU-Net, a framework for biomedical image segmentation that has previously been shown to yield state-of-the-art results for a wide range of public datasets used in international biomedical segmentation competitions (21). The network architecture is a 3D-U-Net, a CNN which is well established in the field of medical image segmentation (24). In the first half of the network, convolutional layers are employed while downsampling the input images at each level, allowing the network to learn features that are relevant at different image resolutions. In the second half, transposed convolutional layers are used to perform an upsampling operation at each level to obtain a volume of same matrix size as the input. The output volume has *N* + 1 channels where each channel represents the predicted probability for each voxel to belong to one of the *N* segmented structures or the background. Finally, a discrete segmentation mask of the image is created by assigning each voxel to the structure with the highest predicted probability. The nnU-Net framework is publicly available (https://github.com/MIC-DKFZ/nnUNet). Settings were as described in the original publication, except from the mirroring data augmentation transform which was disabled to avoid confusion between the left and right cerebellum labels.

The segmentation networks were trained for 1000 epochs where one epoch is defined as an iteration over 250 mini-batches. The mini-batch size was 2. The optimizer was stochastic gradient descent with Nesterov momentum (μ = 0.99). The initial learning rate of 0.01 was decayed during training according to the “poly” learning rate policy (25). The loss function was the sum of cross-entropy and Dice loss (26). In addition, deep supervision was applied in the decoder to all but the two lowest resolutions. Oversampling of foreground classes was applied, guaranteeing that a third of all sampled patches contained one of the foreground classes. The following data augmentation techniques were applied: rotations, scaling, Gaussian noise, Gaussian blur, brightness, contrast, simulation of low resolution and gamma correction.

#### The Classification Network

The pipeline's first classification branch contains a Dense Convolutional Network with 121 trainable layers (DenseNet121) (22). DenseNet's central component is the Dense Block, in which the output of each layer is concatenated to the output of subsequent layers. Compared to preceding network architectures, this improves the information flow and allows for a reduction of network parameters. We used MONAI (version 0.50), a PyTorch-based, open-source framework for deep learning in healthcare imaging for training of the DenseNet and inference (27, 28).

For hyperparameter optimization, the training set was further split into two sets by assigning 12.5% of all cases to a separate hyperparameter tuning set. The network requires as an input a one-hot-encoded segmentation mask (background + VS + adjacent structures). The output is a vector of 4 elements each of which is interpreted as the probability of the VS to be of grade I, II, III or IV.

Input segmentations were reoriented and resampled to a voxel size of 0.8 × 0.8 × 0.8 *mm*^3^ using nearest neighbor interpolation. Data augmentation was applied to artificially increase the size of the training set, using random zooming with factors between 0.9 and 1.1 and a probability of 0.9, random rotations about the axial direction with a probability of 0.8, and by sampling patches of size 256 × 256 × 128 at random positions of the image. An Adaptive Moment Estimation (Adam) optimizer was used with an initial learning rate of 6.1 × 10^−5^ and weight decay of 10^−7^ (29). The loss function was a weighted cross-entropy loss, where the weights are inversely proportional to the number of cases of each Koos grade in the training set.

The training schedule consisted of 100 iterations over all cases in the training set. A batch size of 8 was used. The final model was selected as the one that achieved the highest weighted F1 score on the hyperparameter tuning set.

#### The Koos Random Forest Classifier

Alternatively, we propose another classification branch based on a Random Forest classifier. Random Forests are a popular ML technique for classification and have been shown to be a robust and data-efficient tool. They are collections of decision trees in which each decision tree is based on a random subset of a complete set of features.

##### Feature Extraction

The features fed to Random Forests needs to be carefully designed. Motivated by prior clinical knowledge, three handcrafted features were extracted for each foreground structure of the segmentation mask: volume, shortest distance to the VS and contact area with the VS. The volume *V*_*c*_ of a structure *c* was calculated as *V*_*c*_ = *N*_*c*_ × *V*_*v*_ where *N*_*c*_ represents the number of voxels in the segmentation mask assigned to structure *c*, and *V*_*v*_ is the volume per voxel.

The shortest distance *D*_*c*_ of a structure *c* to the VS was determined by calculating min*D*(*i*_*VS*_, *i*_*c*_), i.e., the smallest distance *D* between any pair of voxels (*i*_*VS*_, *i*_*c*_) where *i*_*VS*_ and *i*_*c*_ represent the voxel indices of voxels of the VS and voxels of structure *c*, respectively.

The contact area *S*_*c*_ of a structure *c* with the VS was calculated in three steps. First, all VS voxels with a neighboring voxel of structure *c* were identified. Secondly, the identified voxels were passed to a marching cubes algorithm [python library Scikit-image (30), version 18.2] which constructs a polygonal surface mesh that represents the part of the VS surface that is in contact with structure *c* (31). Thirdly, the sum of the surface mesh polygon areas was calculated to obtain the total area *S*_*c*_.

All features selected as input for the Random Forest are shown in Table 1.

**Table 1:** Handcrafted features selected as input for the Random Forest classifier.

	**Feature type**		
**Structure**	**Volume**	**Shortest distance to VS**	**Contact surface with VS**
Vestibular schwannoma (VS)	X		
Pons		X	
Brain stem		X	
Cerebellar vermal lobules I-V		X	
Cerebellar vermal lobules VI-VII		X	
Cerebellar vermal lobules VIII-X		X	
Ipsilateral cerebellum		X	X
Contralateral cerebellum		X	

##### Implementation Details

Based on these features a Random Forest is trained and subsequently used to predict the Koos grade of patients whose data was not used in the training process. In this work, we used the Random Forest implementation of the Python library Scikit-learn (32) (version 0.24). Default settings were applied, except for the number of trees of 100,000, maximum tree depth of 5, and minimum samples per leaf of 2.

#### The Ensemble Model

Combining different models in a majority voting ensemble can leverage the strengths of the individual models and boost the overall performance. The proposed ensemble model combines the six classification models obtained from training the DenseNet and the Random Forest three times with the different inputs ceT1, hrT2 and the combination of both modalities. Each of the six models' predictions is counted as a vote for a Koos grade. The ensemble model simply predicts the Koos grade which has the most votes. In case of a possible stalemate between multiple grades, the Random Forest votes were given a casting vote to break the tie.

#### Selection of Relevant Brain Structures

Most of the 160 structures identified by GIF are generally unaffected by the presence of VS so that their features carry little or no relevant information for Koos classification. To identify the most relevant brain structures that need to be segmented for Koos classification, we performed an importance analysis using Random Forest. Specifically, a Random Forest model was trained with the features from all 160 structures. Then, an importance analysis of each feature for classification on a validation set was performed. Unimportant features were removed iteratively until the accuracy reached a maximum. This allowed for identification of the subset of relevant structures to be segmented and used in the subsequent classifications.

The remaining most important features were: volume of the VS, the shortest distances between VS and pons, VS and brainstem, VS and cerebellar vermal lobules I-V, VI-VII and VIII-X, VS and left/right cerebellum white matter, VS and left/right cerebellum exterior, and the contact surfaces between VS and left/right cerebellum exterior. Moreover, the segmentations of the left cerebellum exterior and the left cerebellum white matter were combined into a single segmentation (left cerebellum) without loss of classification accuracy. The corresponding structures on the right side of the brain were combined in the same way. Hence, the final 8 structures are those listed in Table 1. Consequently, the number of input channels of the classification network is 9 (including background).

Furthermore, for the Random Forest, classification performance was found to be improved by converting left and right labels to ipsilateral and contralateral labels with respect to the VS location. To automatically determine whether a tumor was on the left or right side, the MR image and segmentation were reoriented in right-anterior-superior (RAS) orientation. Then, the mean of the projection of the VS segmentation onto the sagittal axis was computed and compared to the mean of the projection of the input MR image. Depending on whether the mean of the projection of the VS was larger or smaller the VS was assumed to be on the right or left side. This approach was found to be robust for all cases in the dataset.

## Experimental Validation

### Metrics

To assess the performances of the different classifiers with respect to the ground truth, three common metrics for multiclass classification were employed: accuracy score, weighted macro-averaged mean absolute error (MA-MAE) and weighted macro-averaged F1 score. In contrast to the accuracy score, the MA-MAE and F1 score take class imbalance into account. Moreover, MA-MAE depends on the difference between true and predicted label, whereas accuracy and F1 scores only consider whether a prediction is correct or not.

The accuracy score is defined as the ratio of the number of correctly classified samples *n*_*match*_ and the total number of samples *n*_*total*_:


$$\begin{array}{l} \text{accuracy}=\;\frac{n_{\text{match}}}{n_{\text{total}}} \end{array}$$

The weighted macro-averaged mean absolute error is defined as (33):


$$\begin{array}{l} \text{MA-MAE}=\;\frac{1}{n}\overset{n}{\underset{j\;=\;1}{\sum }}\frac{1}{n_{j}}\underset{x_{i}\in T_{j}}{\sum }|D\left(x_{i}\right)| \end{array}$$

where *n* represents the number of classes, *n*_*j*_ is the support of class *j* with label *y*_*j*_, i.e., the number of samples belonging to that class, *T*_*j*_ is the set of images in the test set whose true class label is *y*_*j*_, and *D*(*x*_*i*_) is the difference between the true class label and predicted class label for image *x*_*i*_.

The F1 score for binary classification is defined as the harmonic mean of precision and recall:


$$\begin{array}{l} \text{F}1\;=\;\frac{2\;\times \;precision\;\times \;recall}{precision\;+\;recall} \end{array}$$

In multiclass classification, the weighted macro-averaged F1 score is the average F1 score of all classes with weights proportional to the class support.

### Ablation Study and Classifier Comparison

To evaluate the importance of each component of our proposed framework, we perform an ablation study. Firstly, we implement a naïve approach that aims at performing Koos grading using only images (ceT1, hrT2 or ceT1 + hrT2). Secondly, we investigate the benefits of using VS segmentations instead of images for the classification task. Thirdly, we explore the advantages of segmenting the adjacent structures in addition to the VS structure. Finally, we investigate whether adding extra information from the images results in improved performance.

**Figure 3:**
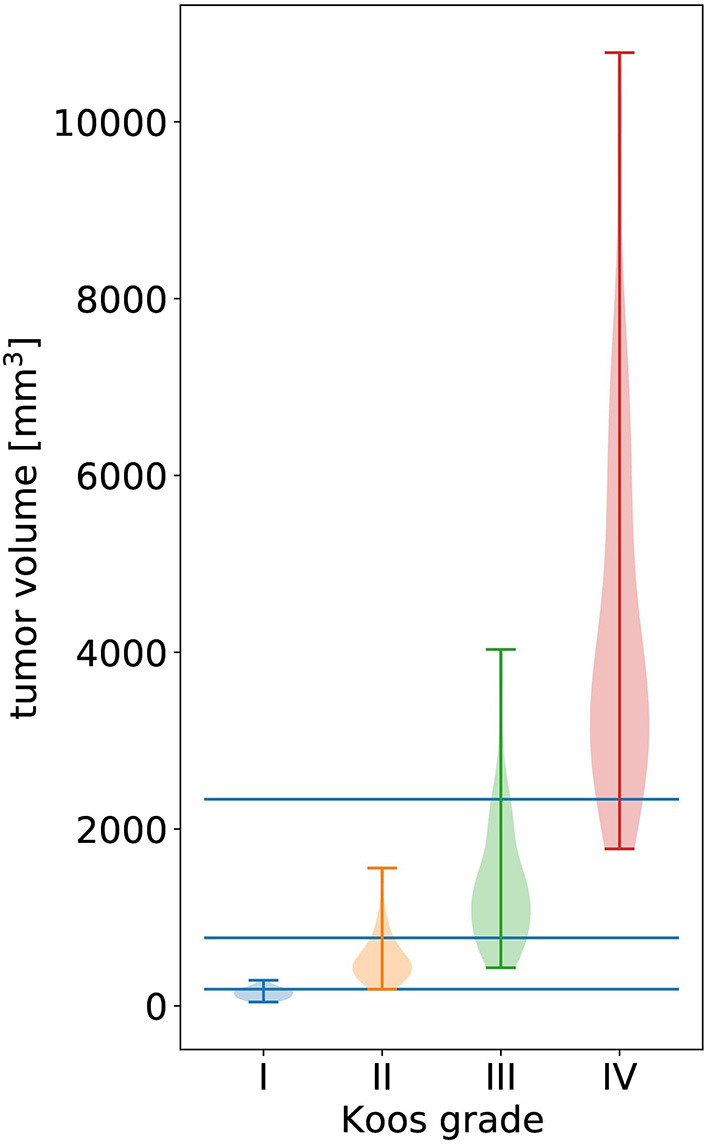
Violin plot of all tumor volumes in the dataset over the ground truth Koos grade. The width of the colored shapes approximately represents the distribution tumor volumes of the Koos grade's samples. The three horizonal lines represent the tumor volume thresholds that optimally separate all samples in terms of weighted F1 score.

Furthermore, we compare the level of performance reached by different types of classifiers. Specifically, we implement two state-of-the-art network architectures [3D DenseNet (22) and 3D EfficientNet (34)] and build the handcrafted features-based Random Forest. Note that we use the DenseNet described in section The classification network and a 3D EfficientNet with a similar number of model parameters (EfficientNet-B3). In addition, we implement a simple baseline algorithm which determines the Koos grade based on learned volume thresholds that optimally separate Koos grades in terms of the weighted F1 score (see Figure 3).

These experiments were evaluated on a test set of 62 cases obtained by stratified random sampling.

### Statistical Tests

Five-fold cross-validation was applied to evaluate and compare the baseline, DenseNet, and Random Forest approach, and the model ensemble to the human annotators. To evaluate the statistical significance of observed differences in the weighted F1 score, a corrected resampled paired *t*-test was applied (35). The correction takes into account that the training sets overlap in a cross-validation approach and are therefore not independent as is assumed in the uncorrected *t*-test. The modified t-statistic is given by: (36)


$$t\;=\;\frac{\bar{d}}{\sqrt{\left(\frac{1}{k}\;+\;\frac{n_{2}}{n_{1}}\right)}\sigma_{d}^{2}}$$

where $\bar{d}$ is the mean difference between paired observations, $\sigma_{d}^{2}$ is its variance, *k* is the number of pairs and $\frac{n_{2}}{n_{1}}$ is the ratio of samples in the test set and training set, e.g., for 5-fold cross-validation $\frac{n_{2}}{n_{1}}=\frac{0.8}{0.2}$.

## Results

### Ablation Study and Classifier Comparison

The results of the ablation study are presented in Table 2. It is apparent that providing only an MRI image as input to DenseNet and EfficientNet is insufficient for accurate classification. Both models show improved performance when the VS segmentation mask with or without adjacent structures is provided instead of the image. The most robust F1-scores for both classifiers are obtained based on the segmentation mask with adjacent structures. Notably, in this case the Random Forest classifier results in the highest scores. Moreover, the DenseNet outperformed the EfficientNet in most experiments. Based on these results, the further experiments were based on the VS segmentation mask with its adjacent structures and classification with Random Forest and DenseNet.

**Table 2:** Comparison of F1-score obtained with different classifiers and with different types of input data and modalities assessed on a single fold of the dataset.

		**F1-score**		
**Input**	**Modality**	**Random forest**	**DenseNet121**	**EfficientNet-B3**
Image	ceT1	–	0.52	0.47
	hrT2	–	0.41	0.37
	ceT1+hrT2	–	0.51	0.31
VS segmentation	ceT1	–	0.86	0.64
	hrT2	–	0.88	0.66
	ceT1+hrT2	–	0.89	0.64
Image +	ceT1	–	0.89	0.76
segmentation of	hrT2	–	0.87	0.65
VS and adjacent	ceT1+hrT2	–	0.75	0.67
structures				
Segmentation of	ceT1	0.94	0.83	0.89
VS and adjacent	hrT2	0.93	0.88	0.88
structures	ceT1+hrT2	0.93	0.90	0.83

### Koos Classification Results

Table 3 shows the 5-fold cross-validation performance of the classification with baseline, DenseNet, and Random Forest approach as well as the human annotators' classification results. The ensemble of DenseNet and Random Forest models achieves the highest scores of all automated methods. When comparing the Random Forest and DenseNet approach, the Random Forest achieves better MA-MAE, weighted F1 score, and accuracy, independent of whether the input modality is ceT1, hrT2 or the combination of both. A large difference can be observed between the two human annotators' scores, with annotator 2 scoring higher than annotator 1. The average score of both annotators is comparable to the score achieved by the ensemble model.

**Table 3:** Koos classification results obtained from automatic methods and human annotators using 5-fold cross-validation.

	**MA-MAE**	**F1 score (%)**	**Accuracy (%)**
**ceT1**			
Baseline	0.23 ± 0.11	76.1 ± 6.3	76.6 ± 5.6
DenseNet	0.17 ± 0.04	81.5 ± 5.1	81.5 ± 5.1
Random forest	0.12 ± 0.05	87.6 ± 3.0	87.6 ± 3.0
**hrT2**			
Baseline	0.22 ± 0.05	79.4 ± 2.1	79.5 ± 2.0
DenseNet	0.15 ± 0.06	83.8 ± 5.8	83.8 ± 5.8
Random forest	0.14 ± 0.06	85.2 ± 4.8	85.2 ± 4.8
**ceT1 + hrT2**			
Baseline	0.22 ± 0.05	77.1 ± 3.7	77.2 ± 3.8
DenseNet	0.18 ± 0.05	82.1 ± 5.0	82.1 ± 5.0
Random forest	0.12 ± 0.06	87.2 ± 2.8	87.2 ± 2.8
**Ensemble**			
DenseNet +	**0.11 ±0.05**	**89.3 ±3.0**	**89.3 ±2.9**
Random forest			
**Human annotators**			
Annotator 1	0.17 ± 0.07	85.4 ± 4.0	84.4 ± 4.7
Annotator 2	0.06 ± 0.02	92.9 ± 3.2	92.9 ± 3.1
Average human annotator	0.11 ± 0.08	89.1 ± 5.2	88.6 ± 5.8

*The columns correspond to the weighted macro-averaged mean absolute error (MA-MAE), the weighted macro-averaged F1 score, and the accuracy score. Inputs are contrast-enhanced T1-weighted (ceT1) images, high-resolution T2-weighted (hrT2) images, or a combination of both (ceT1 + hrT2). The error ranges correspond to the standard deviation of the mean of values obtained from 5-fold cross-validation. Bold scores indicate the best automatic method*.

### Statistical Significance Tests

The *p*-values of the corrected paired *t*-test based on the weighted F1 scores show that both DenseNet and Random Forest are significantly better classifiers than the baseline approach (*p* = 0.042 and *p* = 0.0002, respectively). Moreover, the results of the Random Forest are significantly better compared to DenseNet (*p* = 0.049).

The corrected paired *t*-tests between the ensemble model results and the human annotators across the 5-folds imply that the ensemble model performed significantly better than human annotator 1 (*p* = 0.037), but not significantly different from human annotator 2 (*p* = 0.40). Furthermore, the performance compared to an average human annotator is not statistically significant either (*p* = 0.94).

### Inter- and Intra-rater Reliability

Inter-rater reliability between the two human annotators was assessed by calculating Fleiss' generalized kappa based on 308 cases. The result (k = 0.68), is considered a “substantial agreement” (37). This is in agreement with the inter-rater reliability measured in a recent study (k = 0.71) which was conducted on a much smaller sample size of 40 but with annotations from four human annotators (5).

Intra-rater reliability of human annotator 1 (k = 0.95) and human annotator 2 (k = 0.82) was classified as “almost perfect” according to the weighted kappa metric with quadratic (Fleiss-Cohen) weights. Intra-rater reliability in the aforementioned study was slightly higher with 3 out of 4 annotators achieving “perfect” scores (k = 1.00). One explanation for the lower scores in our study may lie in the increased sample size which made it more difficult for human annotators to recognize the duplicate images and memorize Koos grades assigned to the original images.

### Confusion Matrices

**Figure 4:**
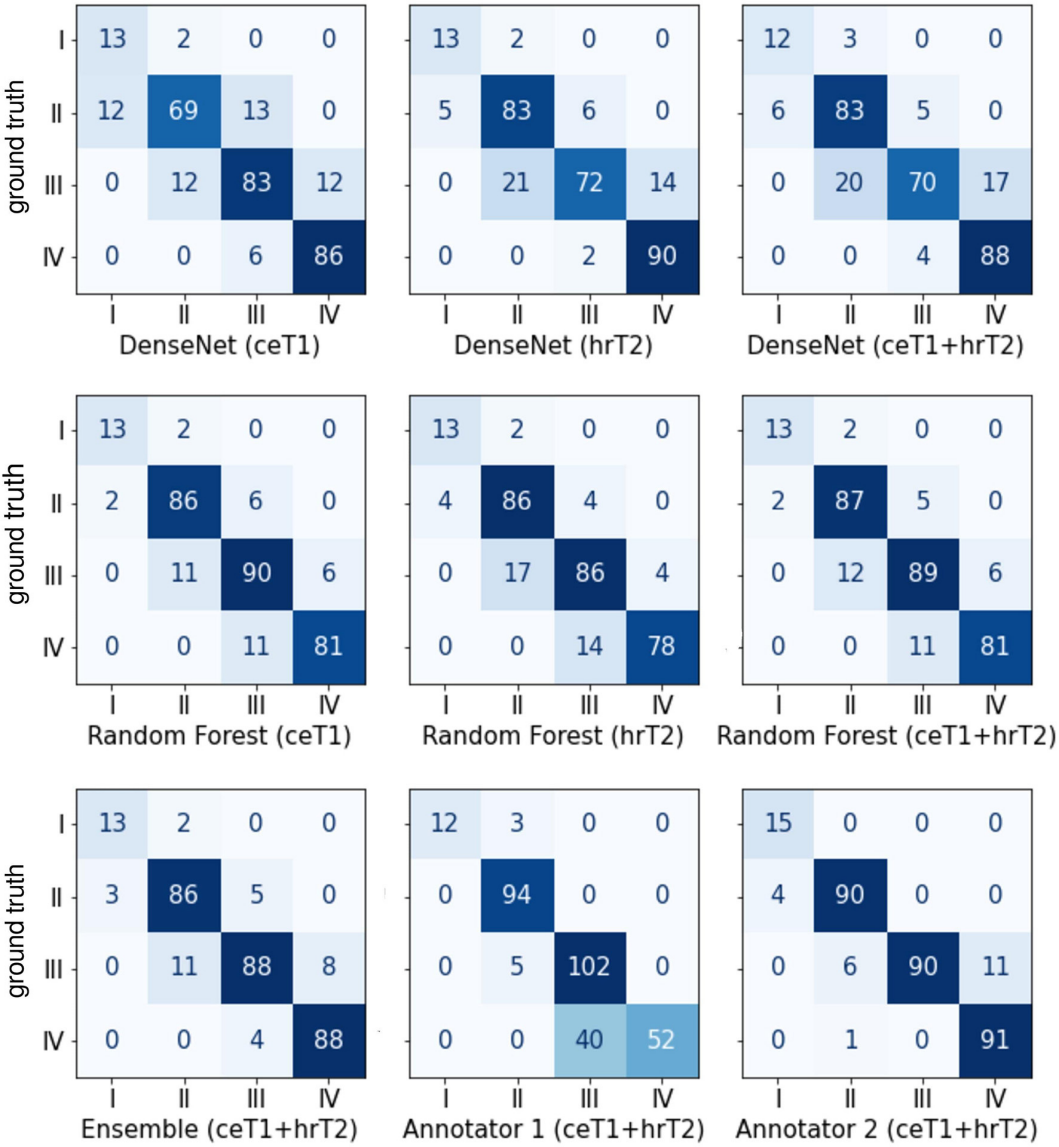
Confusion matrices between the ground truth Koos grades on the horizonal axis and automatic or human Koos grade predictions on the vertical axis.

The confusion matrices shown in Figure 4 give a detailed account of how many cases were correctly classified and how many were misclassified for each Koos grade. They also reveal by how many grades misclassified cases were separated from the ground truth.

Notably, none of the classifiers misclassified a case by more than one grade, except for human annotator 1 who classified a grade IV tumor as grade II.

Moreover, it is apparent that the majority of misclassifications by human annotators are between Koos grades III and IV whereas for the automated approaches the number of misclassifications is more evenly spread over all grades. For example, human annotator 1 classified 40 cases as III instead of IV. In contrast, both human annotators outperform the automated pipelines when only Koos grades I-III are considered.

### Segmentation Results

To assess the quality of the intermediate segmentations predicted by nnU-Net in the first stage of the pipeline, Dice scores were calculated for all structures (Table 4). Excellent results with average Dice scores across all segmented brain structures of 94.8 ± 1.4%, 92.4 ± 1.5%, and 94.8 ± 1.4% were obtained for ceT1, hrT2, and ceT1 + hrT2 inputs. Dice scores are generally higher for ceT1 and ceT1 + hrT2 inputs than for hrT2 only inputs, respectively. For pons and brainstem, the highest Dice scores are achieved, whereas cerebellar vermal lobules are more difficult to segment and achieve lower Dice scores for all input modalities.

**Table 4:** Results of the automatic segmentation of selected brain structures.

	**Dice score (%)**		
**Structure**	**ceT1**	**hrT2**	**ceT1 + hrT2**
VS	93.9 ± 4.0	90.7 ± 5.4	94.1 ± 4.1
Pons	97.6 ± 0.8	96.7 ± 0.9	97.6 ± 0.8
Brainstem	96.3 ± 1.3	94.4 ± 1.3	96.3 ± 1.2
Cerebellar vermal lobules I-V	93.2 ± 2.3	89.3 ± 2.3	93.2 ± 2.3
Cerebellar vermal lobules VI-VII	87.8 ± 5.1	84.0 ± 5.1	87.8 ± 5.1
Cerebellar vermal lobules VIII-X	93.1 ± 2.3	89.3 ± 2.3	93.1 ± 2.3
Right cerebellum	95.8 ± 1.4	93.9 ± 1.5	95.8 ± 1.4
Left cerebellum	95.8 ± 1.5	93.8 ± 1.6	95.8 ± 1.5

*The segmentation model trained with nnU-Net was compared to the ground truth segmentations. Inputs are contrast-enhanced T1-weighted (ceT1) images, high-resolution T2-weighted (hrT2) images, or a combination of both (ceT1 + hrT2). The error ranges correspond to the standard deviation of the mean Dice score of all test cases*.

Note that our segmentation model (Dice scores based on ceT1/hrT2/ceT1 + hrT2 = 93.9 ± 4.0/90.7 ± 5.4/94.1 ± 4.1) compares favorably with the state-of-the-art model (9) for VS segmentation trained on the same dataset (Dice scores based on ceT1/hrT2/ceT1 + hrT2 = 93.43 ± 3.97/88.25 ± 3.90/93.68 ± 2.80).

## Discussion

In this work, we present the first automated methods for classifying VS tumors according to their Koos grade. The best classification performance was obtained with the model ensemble which leverages the strengths of the two proposed pipelines and modalities, and achieves results that are comparable to those of a human annotator.

### Learned Features vs. Handcrafted Features

We observe that deep learning-based models are outperformed by a standard ML approach designed with handcrafted features. This could be explained by the lack of sufficient training data since neural networks require more data for optimal performance. While the number of training cases in the dataset is relatively large for the segmentation task, it can be considered small for the classification step of the pipeline. This is due to the larger amount of information contained in a segmentation label compared to the single scalar label provided for each image in the case of classification. On the one hand, by extracting handcrafted features and discarding all other information from the segmentation, a simpler classification task is constructed which requires fewer training samples and a less complex classifier (Random Forest). On the other hand, although the handcrafted features were carefully selected some possibly relevant information, for example the shape of the tumor, is lost in the feature selection process. Manual feature selection therefore represents a trade-off between focus on relevant features and consideration of less relevant features.

In settings in which only one modality is available, the Random Forest method is recommended as this classifier yields better results than DenseNet and is more robust to different random initializations of model parameters. In addition, the time required for training the Random Forest model (<2 min) is short compared to that of the DenseNet model (~4 h).

On the other hand, the DenseNet has a much shorter inference time (<1 s) than the Random Forest branch of the inference pipeline where the bottleneck is the extraction of handcrafted features (~5 min), although the latter estimate is expected to drop drastically if parallelized versions of the feature extraction algorithms are employed. Furthermore, we expect the DenseNet approach to benefit from a larger number of images in the training set, since neural networks tend to require larger amounts of data than traditional machine learning algorithms. Koos grade I, in particular, is underrepresented in this dataset (12 training cases), but even the sample sizes of the other grades (75–85 training cases per grade) are considered relatively small for classification tasks with deep learning.

### Classification Using an Ensemble

It can be observed that the use of a model ensemble leads to a boost in performance. This could be explained by the fact that the ensemble favours the model that is more certain in its prediction across the three types of input modality. For example, the shape of a small tumor might clearly indicate a protrusion of the tumor outside the meatus and be correctly classified across all input types only by the DenseNet. On the other hand, due to the small number of training data DenseNet might not learn that contact between tumor and brain stem is a decisive feature between Koos grade II and III. This, however, is easier to learn for the Random Forest based on the provided distance features between tumor and adjacent structures. The ensemble model can predict the correct grade in both examples.

A disadvantage of the model ensemble is that it requires the availability of co-registered images of both modalities that are resampled in the same voxel space. Moreover, both pipelines need to be set up and three models trained for each of them (ceT1, hrT2, and ceT1 + hrT2).

### Impact of Input Modalities

The notably worse classification results observed for hrT2 only inputs can be attributed to the lower segmentation quality of the first neural network for all segmented brain structures (Table 4). Due to the reduced image contrast between VS and adjacent brain structures the identification of tumor boundaries is often more challenging which explains the reduction in Dice score. Nevertheless, interest in non-contrast images has increased in recent years in an effort to reduce patient risk and scan cost (38). Gadolinium-based contrast agents have indeed been associated with risks such as accumulation in the brain and nephrogenic systemic fibrosis in patients with impaired renal function (39, 40). Hence, research into exploiting hrT2 images for assessment of VS is ongoing and has led to T2-weighted imaging becoming a standard approach for initial detection and follow-up of untreated VS (41).

### Analysis of Misclassifications

**Figure 5:**
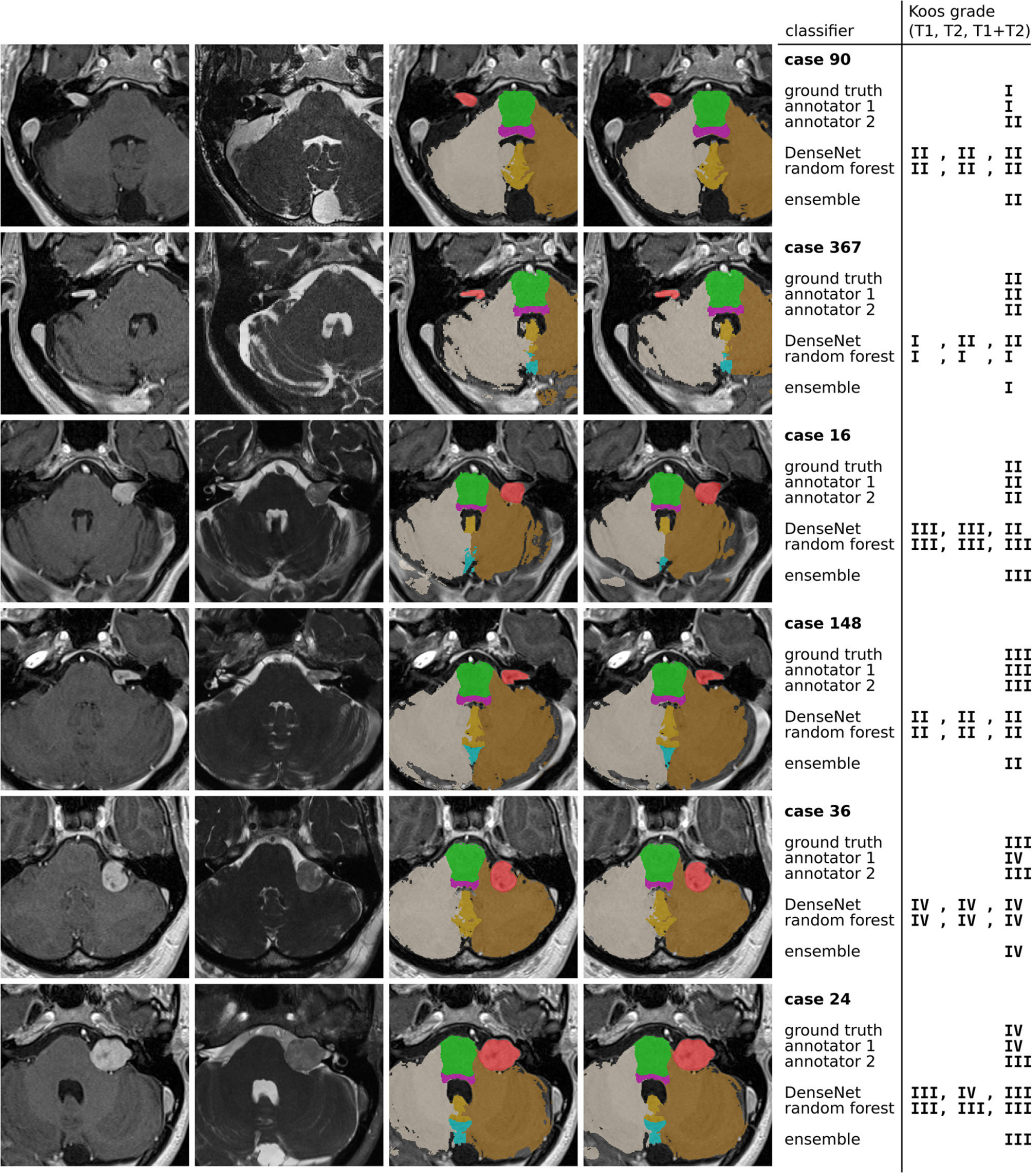
Examples of misclassified cases. The 4 columns contain ceT1 images, hrT2 images, ground truth segmentations resulting from GIF, and predicted segmentations by the nnU-Net model. The colors indicate the segmentation labels of VS (red), pons (green), brain stem (purple), left cerebellum (brown), right cerebellum (gray), cerebellar vermal lobules I-V (yellow) and VI-VII (light blue).

Figure 5 highlights cases that were correctly classified by at least one human annotator and misclassified by the ensemble model.

The first and second row show a grade I and grade II tumor, respectively. The tumor is particularly large for a grade I tumor, but it does not protrude beyond the porus of the petrous bone into the cerebellopontine angle (most evident on the hrT2 image). In contrast, the grade II tumor has a smaller volume, however, there is clear protrusion of the medial extrameatal component into the cerebellopontine angle.

A similar situation is presented in row 3 and 4, which show a large grade II and a relatively small grade III tumor, respectively. While the human annotators agree with the ground truth, all automatic models except for one confuse the Koos grades. The decisive feature for the classification of the grade II tumor is the increased intensity in the hrT2 image of voxels at the border between VS and cerebellum, indicating a CSF filled cleft between both structures. This feature is not present in the hrT2 image of the grade III tumor. However, based on only the ceT1 image or the segmentations this distinction is hardly possibly.

Finally, rows 5 and 6 present cases for which the models, and one human annotator confuse Koos grade III and IV. Both cases exhibit a degree of brain stem compression, however the compression is more pronounced in the grade IV case.

For human annotators, the distinction between these two grades was difficult across much of the dataset as can be seen in the confusion matrices of Figure 4. Annotator 1 misclassified many grade IV cases as grade III whereas annotator 2 misclassified grade III cases as grade IV. This suggests that both annotators interpreted the meaning of “brain stem compression” in the Koos scale description differently with annotator 2 frequently labeling cases where there was only cerebellar peduncle compression (and no brainstem compression) as Koos grade IV. This discrepancy highlights a shortcoming of the Koos scale itself. A clearer definition of which brain structures are considered part of the brain stem and which degree of compression determines the transition from grade III to grade IV would likely increase inter-rater reliability. The presented examples highlight borderline cases that depend on nuances in the MR images and on the interpretation of the Koos scale which ultimately limit the accuracy that a classifier can achieve.

### Limitations

This study was limited by its rather homogeneous dataset which was acquired for GK therapy guidance with a standardized scan protocol on a single MRI scanner. Therefore, the question of how well the suggested pipeline generalizes on diagnostic datasets typically acquired on different scanners and with a wide range of values for scan parameters such as image resolution, field of view and sequence type remains open.

However, we believe that the 2-stage design of our pipeline makes it possible for it to generalize well to heterogeneous datasets. This is because the framework applied in the first stage, nnU-Net, was specifically developed to adapt and automatically configure itself for any new dataset. Our preliminary results (not shown here) on a clinical dataset of MR images of patients with VS acquired on different scanners and with different scan protocols show that nnU-Net is capable of producing highly accurate segmentations of VS and adjacent structures for such heterogeneous datasets.

The second stage of our pipeline depends exclusively on the output segmentations produced by nnU-Net. Barring segmentation errors, these segmentations are independent of the scan protocol or modality since they represent the morphology of brain structures; thus, the second stage models (DenseNet and Random Forest) are not expected to require image-specific adaptation. On the other hand, the exclusive dependence of the classifier on the segmentation output also means that an inaccurate segmentation output is likely to result in misclassification.

The pipeline's second limitation is that the GIF algorithm is not optimized for T2-weighted images or for images with a restricted field of view (FOV), e.g., a FOV focused only on Internal Auditory Meatus. Currently, T2-weighted images in the training set need to be accompanied by co-registered T1-weighted images which are used as input for GIF.

Finally, the dataset includes a relatively small number of Koos grade I cases since such tumors are rarely treated with GK SRS. Diagnostic datasets are usually more evenly distributed across all Koos grades. In addition, such datasets typically include tumors that are too large for radiosurgery which are not present in the current curated GK dataset.

### Future Work

In future work, we aim to address these limitations by evaluating the complete pipeline on non-curated datasets obtained on a variety of clinical scanners with varying sequence protocols. Furthermore, while this study focuses on sporadic VS, adaptations of the pipeline for MR images of bilateral tumors from patients with the hereditary condition Neurofibromatosis type 2 (NF2) will be developed. Finally, we aim to combine the automatically predicted Koos grades with other frequently reported tumor measures to investigate how their availability might facilitate, on the one hand, the preparations for a multidisciplinary team meeting, and on the other hand, the treatment decision process during the meeting itself.

## Conclusions

We developed the first framework for fully automated Koos classification. The excellent results on ceT1 and hrT2 images show that the classification accuracy is comparable to that of neurosurgeons. Further validation on clinical datasets can enable the framework to be applied as a support tool for treatment planning in clinical practice.

In combination with other tumor characteristics, such as the largest extrameatal diameter and the tumor volume, the Koos scale is routinely employed in clinical practice to facilitate treatment planning and patient counseling. In clinical practice, it has the potential to increase the efficiency of treatment planning by reducing preparation time and workload. In the future, further developments of the pipeline could aim at the automated calculation of other tumor characteristics such as the extrameatal diameter to provide a complete set of relevant metrics to the treatment team.

## Data Availability Statement

Publicly available datasets were analyzed in this study. This data can be found here: The Cancer Imaging Archive (TCIA) (https://doi.org/10.7937/TCIA.9YTJ-5Q73).

## Ethics Statement

The studies involving human participants were reviewed and approved by NHS Health Research Authority and Research Ethics Committee (18/LO/0532). Written informed consent for participation was not required for this study in accordance with the national legislation and the institutional requirements.

## Author Contributions

This study was conceived and designed by AK, RD, TV, and JS. The experiments were performed by AK and RD. The clinical dataset was acquired by DG and IP. Data were analyzed and annotated by SC, AO, MO, NK and JS. The results were discussed by all authors. The manuscript was written by AK, RD, and JS. All authors read and approved the final version of the manuscript.

## Funding

This work was supported by Wellcome Trust (203145Z/16/Z, 203148/Z/16/Z, WT106882), EPSRC (NS/A000050/1, NS/A000049/1), and MRC (MC/PC/180520) funding. TV was supported by a Medtronic/Royal Academy of Engineering Research Chair (RCSRF1819/7/34).

## Conflict of Interest

SO is co-founder and shareholder of BrainMiner Ltd., UK. The remaining authors declare that the research was conducted in the absence of any commercial or financial relationships that could be construed as a potential conflict of interest.

## Publisher's Note

All claims expressed in this article are solely those of the authors and do not necessarily represent those of their affiliated organizations, or those of the publisher, the editors and the reviewers. Any product that may be evaluated in this article, or claim that may be made by its manufacturer, is not guaranteed or endorsed by the publisher.
